# Editorial: Effects of pathogen parasitism on host metabolism in aquaculture animals

**DOI:** 10.3389/fcimb.2024.1467352

**Published:** 2024-09-20

**Authors:** Rongrong Ma, Lei Liu, Raid Alrowais, Ya-Xiong Tao, Yibin Yang, Renyuan Li

**Affiliations:** ^1^ School of Marine Sciences, Ningbo University, Ningbo, China; ^2^ Department of Civil Engineering, College of Engineering, Jouf University, Sakakah, Saudi Arabia; ^3^ School of Veterinary Medicine, Auburn University, Auburn, AL, United States; ^4^ Yangtze River Fisheries Research Institute, Chinese Academy of Fishery Sciences, Wuhan, China

**Keywords:** microorganism, pathogens, host metabolism, interaction, aquaculture

Aquaculture serves as a sustainable food source, substantially contributing to global fish production. Like other agricultural activities, aquaculture is vulnerable to diseases that can threaten the health and productivity of farmed species. By exploring the interactions between aquatic pathogens and their hosts, we can gain some basic information about therapeutic and preventive strategies for aquaculture animal diseases from a metabolic perspective.

It is our pleasure to present the Research Topic “Effects of pathogen parasitism on host metabolism in aquaculture animals” to the readers. This Research Topic is dedicated to exploring the impact of pathogen parasitism on the aquaculture animals. This topic collects five original research articles that encompass epidemiological investigation of pathogen parasitism, treatment of disease, environmental and gut microbiome, and therapeutic strategies targeting the metabolic pathways of pathogenic bacteria.

An epidemiological investigation of *Larimichthys crocea* diseases in the Ningbo area was conducted (Xu et al.). A total of 55 dying *L. crocea* with obvious clinical symptoms were collected, 78.18% of which were detected to be caused by pathogenic infection. Among them, 25 strains of pathogenic bacteria were isolated, primarily *Pseudomonas plecoglossicida* and *Vibrio harveyi*. Additionally, two parasites, *Cryptocaryon irritans* and *Neobenedenia girellae*, were observed. The red sea bream iridovirus (RSIV) was the predominant detected virus. This investigation evaluated the risk factors of the disease in *L. crocea*. The study on the high efficacy of copper plates against the tomont of *C. irritans* offers valuable information for the prevention and control of *C. irritans* in the practice of aquaculture (Guo et al.). This study offers valuable insights into the potential mechanism of action of *C. irritans* tomonts under copper plate stress.

Seasonal variations in the gut microbiota of *Eriocheir sinensis* demonstrate the intricate relationships between the gut microbiota and the host environment. The findings revealed the complexity of the microbial interaction network in different seasons (Qin et al.). Additionally, investigating the microorganisms in the guts of *Exopalaemon annandalei* and *Exopalaemon carinicauda* can help us understanding the survival status of shrimps in the Yangtze River estuary. The dominant bacteria in the gut flora of these two shrimps belonged to the phyla Proteobacteria and Firmicutes, respectively. The key potential functions of the gut microbiota include amino acid metabolism and purine metabolism, providing a theoretical basis for understanding the gut bacterial communities of estuarine shrimp and their healthy aquaculture (Wang et al.). In addition, metabolism-based therapies for aquaculture organisms hold promise in optimizing their health and performance by targeting specific metabolic pathways that are impacted by pathogens or environmental stressors. The therapeutic effect of glutathione on abnormal liver lipid metabolism provides a scientific basis for the healthy development of the bullfrog industry by regulating multiple metabolic pathways, and alleviating disorders in glycerophospholipid and amino acid metabolism (Su et al.).

Overall, pathogen prevalence, host characteristics, drug prevention and control, environmental impacts, and metabolic pathways were explored. Their results can provide important information for the prevention and control of aquaculture disease from multiple angles based on the direction of metabolism. We hope and expect that the aquaculture researchers will find this Research Topic of articles helpful. As editors, we would like to appreciate the authors for their dedications to the topic. We would also like to convey our gratitude to all referees for their careful evaluation of the articles. Many thanks to all the colleagues who responded to this call, but whose interests could not be accommodated within the confines of this Research Topic. Finally, we extend our sincere appreciation to Frontiers in Cellular and Infection Microbiology for supporting this exciting Research Topic.

